# Characterization of Cortical Neuronal and Glial Alterations during Culture of Organotypic Whole Brain Slices from Neonatal and Mature Mice

**DOI:** 10.1371/journal.pone.0022040

**Published:** 2011-07-15

**Authors:** Jerome A. Staal, Samuel R. Alexander, Yao Liu, Tracey D. Dickson, James C. Vickers

**Affiliations:** Menzies Research Institute, University of Tasmania, Hobart, Tasmania, Australia; Virginia Commonwealth University, United States of America

## Abstract

**Background:**

Organotypic brain slice culturing techniques are extensively used in a wide range of experimental procedures and are particularly useful in providing mechanistic insights into neurological disorders or injury. The cellular and morphological alterations associated with hippocampal brain slice cultures has been well established, however, the neuronal response of mouse cortical neurons to culture is not well documented.

**Methods:**

In the current study, we compared the cell viability, as well as phenotypic and protein expression changes in cortical neurons, in whole brain slice cultures from mouse neonates (P4–6), adolescent animals (P25–28) and mature adults (P50+). Cultures were prepared using the membrane interface method.

**Results:**

Propidium iodide labeling of nuclei (due to compromised cell membrane) and AlamarBlue™ (cell respiration) analysis demonstrated that neonatal tissue was significantly less vulnerable to long-term culture in comparison to the more mature brain tissues. Cultures from P6 animals showed a significant increase in the expression of synaptic markers and a decrease in growth-associated proteins over the entire culture period. However, morphological analysis of organotypic brain slices cultured from neonatal tissue demonstrated that there were substantial changes to neuronal and glial organization within the neocortex, with a distinct loss of cytoarchitectural stratification and increased GFAP expression (p<0.05). Additionally, cultures from neonatal tissue had no glial limitans and, after 14 DIV, displayed substantial cellular protrusions from slice edges, including cells that expressed both glial and neuronal markers.

**Conclusion:**

In summary, we present a substantial evaluation of the viability and morphological changes that occur in the neocortex of whole brain tissue cultures, from different ages, over an extended period of culture.

## Introduction

The exciting development of long-term brain slice cultures has revolutionized many experimental studies by providing an ideal platform between dissociated cell cultures and *in vivo* studies. Accordingly, organotypic brain slice cultures have been used, for example, as models for stroke [1], [2], epilepsy [3], [4], neuronal injury [5], and neuroprotection [4], [6]. The recent development of substrates with microelectrode arrays has further permitted functional assays of brain slice activity and electrophysiological changes in these various pathological conditions [5], [6].

There have been two principle techniques for culturing brain slices: the roller tube method and the membrane interface method [6]. Gahwiler was among the first to develop the roller-tube method, which involved embedding the tissue in either a plasma clot or in a collagen matrix on glass coverslips followed by continuous slow rotation [7]. This organotypic technique often results in the flattening of slices into a quasi-monolayer, providing a useful platform for microscopic imaging and easy manipulation of individual living neurons [8]. Alternatively, the membrane interface method is also advantageous as it is a relatively simple method and the cultures retain a semi-three-dimensional structure. This is particularly useful for morphological studies, electrophysiological techniques and/or biochemical studies [9], [10], [11].

Characterization of brain slice organotypic cultures has mainly been conducted with respect to studies of the hippocampus, as these culture preparations form the ideal model for synaptic studies [6], [11], [12]. However, there remains a distinct lack of studies into the cortical neuronal changes that occur following the culture of whole brain slices, particularly in mice. Given the important role of cortical neuronal pathways both in disease and after injury, whole brain slice cultures containing the cerebral cortex would be particularly advantageous. Moreover, the tremendous advances in the development of transgenic mouse models would provide a further experimental model to complement these organotypic slice techniques. The majority of the organotypic slice culture models involve tissue from neonates that are maintained for an extended period of between 7 to 16 days *in vitro* (DIV). Neonates (from P0–P8) are ideally suited to culturing as they are proposed to survive explantation more readily [8]. In this regard, only a few studies have attempted to culture brain slices from more mature brain tissue [6], [13], [14], [15]. Organotypic hippocampal cultures from young adult rats has proved to be a particularly valid model in the *in vitro* study of brain injury, and is particularly advantageous as these long term (7DIV) cultures maintain tight organization of neuronal layers [13]. At least in hippocampal slices, organotypic cultures prepared from young adults also maintain synaptic activity *in vivo*, in contrast to cultures from peri-natal animals that sometimes display aberrant synaptic activity after long-term culture [16]. It is unclear what cellular and cytoarchitectural distortions occur in the cerebral cortex during the development of neonatal tissue in culture, as key cortical neuronal populations are still in their migratory phase [17]. It is therefore important to consider the interpretation of data from such organotypic models derived from early development for their relevance to experimental studies related to diseases and conditions that affect the mature nervous system.

In this study, we investigated cell survival as well as the cellular and protein expression alterations that occur during the culture of whole brain slice cultures from neonates, young animals and mature adult mice.

## Materials and Methods

### 2.1 Ethics statement

All animal procedures were performed in accordance with and approved by the animal ethics guidelines of the University of Tasmania Animal Ethics Committee (ethics approval number A9398). Strict attention was given to the care and use of animals according to Australian Law. Animals euthanized by injection of sodium pentobarbitone, followed by cervical dislocation (in adult animals) or decapitation (neonates).

### 2.2 Organotypic brain slice preparation

Organotypic brain slices were prepared according to the membrane interface method described by De Simoni and Yu [9], with minor modifications. Briefly, C57BL/6 mice pups (n = 32) of post-natal day 4–6 (P4–6), juveniles (n = 26) of P25–28 and mature adults (n = 26) of P50+ were sacrificed and immediately decapitated according to the procedures approved by the institutional animal care committee. For neonates, mice were placed in ice for 5–6 min, whilst mature animals were anaesthetized with sodium pentobarbitone (140 mg/kg sodium pentobarbitone, i.p. Virbac, Peakhurst, Australia) prior to decapitation. After decapitation, the brains were rapidly removed, and medial sections of the brain isolated (Fig. S4). Coronal slices were obtained in ice-cold using a Leica VT1000E vibratome® according to previously reported [9]. The entire dissection was conducted in ice-cold slicing medium consisting of Earles balanced salt solution (EBSS; Gibco-Invitrogen) and HEPES (Sigma) as detailed previously [9]. For neonates, brain slices were cut at 400 µm thickness whilst mature animals were cut at 250 µm. Suitable slices were transferred to sterile, humidified semi-porous Millex-GP inserts with a pore size of 0.22 µm (2–3 slices/insert; Millipore). The inserts were prepared 2 hrs prior to culture by placing them in 6-well culture trays (Falcon, VWR, Canada) containing culture medium consisting of 50% MEM with Glutamax-1 (Gibco), 25% EBSS, 25% heat inactivated horse serum (Gibco), 35 mM glucose (Sigma), nystatin (Sigma) and penicillin-streptomycin (Gibco). Slices were maintained at 37°C in 5% CO_2_ humidified incubator, with complete media changes done 1 day after plating then twice a week subsequently. Acute slices from each animal, brain slices that were not placed on inserts, were allowed to recover for 60 min in 4°C slicing solution and used as controls for cell viability assays. Importantly, acute brain slices were used to represent the state of brain slices prior to culture.

### 2.3 Cell viability assays

Cell damage was detected using the exclusion dye propidium iodide (PI; Molecular Probes, UK) as previously described [18]. Briefly, at 4, 7 or 14 DIV cultures were placed in fresh culture medium containing 5 µg/ml of PI for 30 min and then imaged using an inverted Leica DMIL epifluorescence microscope fitted with standard rhodamine optics (excitation 510–560 nm; dichroic mirror 620 nm; emission >590 nm). Images of the entire cultured slice were taken using a Hamamatsu (ORCA-ER) digital camera at 5× (numerical aperture 0.12) magnification. Similarly, acute brain slices were also incubated with 5 µg/ml of PI for 30 min and imaged, providing baseline for cell damage occurring at time of culture. Incubation of acute slices with PI was conducted in a similar manner to that seen with organotyptic brain slice cultures (i.e. after resting acute slices for 60 min as per protocol, slices were placed on membrane inserts and placed into medium containing PI). Images of these slices were taken using an inverted Leica DMIL epifluorescence microscope (Figs. S1, S2, S3, and S4). PI labeled cells, those above a pre-defined fluorescence threshold [19], in organotypic slices were counted (double-blinded) across the entire field and counts were expressed as a percentage of that calculated in acute slices from animals of the same age. Statistical analysis was performed using a one-way ANOVA with Bonferroni's multiple comparison post-test where significance was considered to be having a p value<0.05.

Neuronal respiration (metabolism) was assessed using alamarBlue® rapid and accurate cell health indicator (Molecular probes). The AlamarBlue® assay is quantative in respect to both dose and time, utilizing the ability of metabolically active cells to convert the reagent into a fluorescent and colorimetric indicator [20]. A 1∶10 dilution of the AlamarBlue® mix-and-read solution was incubated with the slice cultures for 1 hr at 4, 7, 14 DIV. After incubation, the absorbance of the sample solution was read on a UV-Vis spectrophotometer at 570 nm.

### 2.4 Immunohistochemistry

For immunohistochemical analysis, brain slice cultures were cryoprotected in 18% then 30% sucrose solutions, embedded in Shandon cryomatrix tissue compound (Thermo Scientific, Runcorn, UK) and sub-sectioned on a cryostat at thickness of 40 µm directly onto Shandon colorfrost Plus slides (Thermo Scientific). It is suggested that cells within an organotypic brain slice culture (interface method) can re-organize and stratify so that neurons migrate to the bottom of the culture and a glial “cap” forms on the exposed upper surface [21]. For this reason, subsections were serially sliced to ensure that only the sections of culture at the membrane-medium interface were utilized. The slides were gently washed three times in PBS, prior to antigen heat retrieval (citrate buffer pH 9; 10 min in electric pressure cooker). Sections were blocked by treatment with DAKO Protein Block, Serum-Free (DAKO, ON, Canada) for 1 hr prior to incubation overnight in a solution containing antibodies against either glial fibrillary acid protein (GFAP, 1∶5,000, rabbit polyclonal; Abcam), a neuronal cell-marker [22] SMI 312 (1∶2500, mouse monoclonal; Sternberger Monoclonal Inc.), and neurofilament light subunit (NFL) (1∶2000, rabbit polyclonal; Millipore) prepared in diluent (0.1% PBS, 0.03% Triton X-100™). Sections were then washed and incubated with secondary antibodies targeting either goat anti-mouse IgG conjugated to Alexafluor 488 (1∶10 000; Molecular Probes) or goat anti-rabbit IgG conjugated to Alexafluor 594 (1∶10 000; Molecular Probes) for 2 hrs [23]. Slices were also stained with Nuclear Yellow (Hoescht S76912, Invitrogen) for 15 minutes (1 µg/ml) prior to washing and placing on slides. Images were collected on an upright epifluorescence Leica DMLB2 microscope fitted with an Optronics Magnafire cooled CCD camera.

### 2.5 Western Blotting

Immunoblotting studies were performed as described previously [24], slices (n = 9 slices/DIV/dissection) were washed with cool PBS and transferred from the membrane inserts to 300 µl lysis buffer (10 mM Tris, 25 mM EDTA, 100 mM NaCl, 1% NP-40, 1% Triton X-100, one tablet Roche® protease inhibitor cocktail, pH 7.35). Samples were homogenized manually over ice followed by sonication, and centrifuged at 13,000 rpm for 5 min. Protein concentration was determined with the BCA protein kit (Pierce, Rockford, IL) using bovine serum albumin as a standard. Homogenates were resolved by sodium dodecyl sulfate-polyacrylamide gel electrophoresis (SDS-PAGE) (8% or 10% polyacrylamidegels) according to Laemmli [25], and transferred to PVDF (Millipore, Inc.). The membranes were incubated with 5% nonfat dry milk in TBST at 4°C for 60 min and probed overnight with primary antibodies to glial fibrillary acid protein (GFAP, 1∶1,000, rabbit polyclonal; Abcam), synaptophysin (1∶1,000, mouse monoclonal; Abcam), and growth-associated protein-43 (GAP-43,1∶1,000, mouse monoclonal; Abcam) at 4°C overnight. Immune complexes were detected by enhanced chemiluminescence with anti-mouse or anti-rabbit immunoglobulin G coupled to horseradish peroxidase as the secondary antibody (Amersham-Pharmacia). To ensure the equal loading and accuracy of changes in protein abundance, the protein levels were normalized to Glyceraldehyde 3-phosphate dehydrogenase (GADPH) abundance. The abundance of GADPH closely paralleled the amount of total protein loaded and was used to normalize the measured relative density of each band of interest within a linear range of exposures (ImageJ, NIH). Protein expression change was normalized to the first time-point in culture as reported previously [12], however, no statistical analysis was made between cultured from different age groups.

## Results

### 3.1 Organotypic brain slice viability

**Figure 1:**
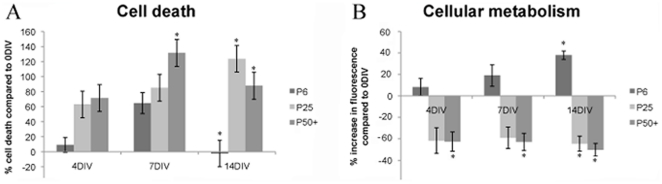
Graphs representing cell death and metabolism of organotypic brain slice cultures. *A*; Cell death was assessed using propidium iodide labeling of nuclei material which occurs due to compromised cell membranes. *B*; Cellular metabolism was assessed using the cell viability assay alamarBlue®. Both cell death and metabolism are expressed as a percentage of that measured in acute brain slices (0 DIV).

Two different cell viability assays were utilized in this study to determine brain slice health during the entire culture period. Comprised cellular membranes (may be indicative of necrosis) were determined using PI staining, as widely reported in many brain slice experimental studies [18], [26], [27]. The percentage change in cell death (which we determined as cells having PI labeling) compared to acute brain slices (0 DIV) were significantly (p<0.05) reduced over 14 DIV in brain slices cultures from P6 mice (Fig. 1A). Brain slice cultures from P25 mice, however, showed a significant increase in cell death at 14 DIV with no reduction in apoptotic nuclei observed during the entire culture period (Fig. 1A). Comparably, slices from P50+ mice displayed significantly higher levels of cell death earlier (7 DIV) than that in slices from younger animals.

AlamarBlue ® is a rapid and accurate reagent for evaluating cellular health by indirectly determining innate metabolic activity [20]. In P6 mice, there is a gradual and significant increase in metabolic activity over the 14 DIV (Fig. 1B). This may be indicative of proliferation of cells in culture or increased metabolic function [20]. Organotypic brain slices from more mature animals, however, have reduced metabolic activity during the entire culture period with significant (p<0.05) decreases in metabolic activity compared to acute (0 DIV) brain slices at 14 DIV in cultures from P25 mice, as well as at 4, 7 and 14 DIV in slice cultures from P50+ mice.

### 3.2 Culture-induced neuron morphological alterations

**Figure 2:**
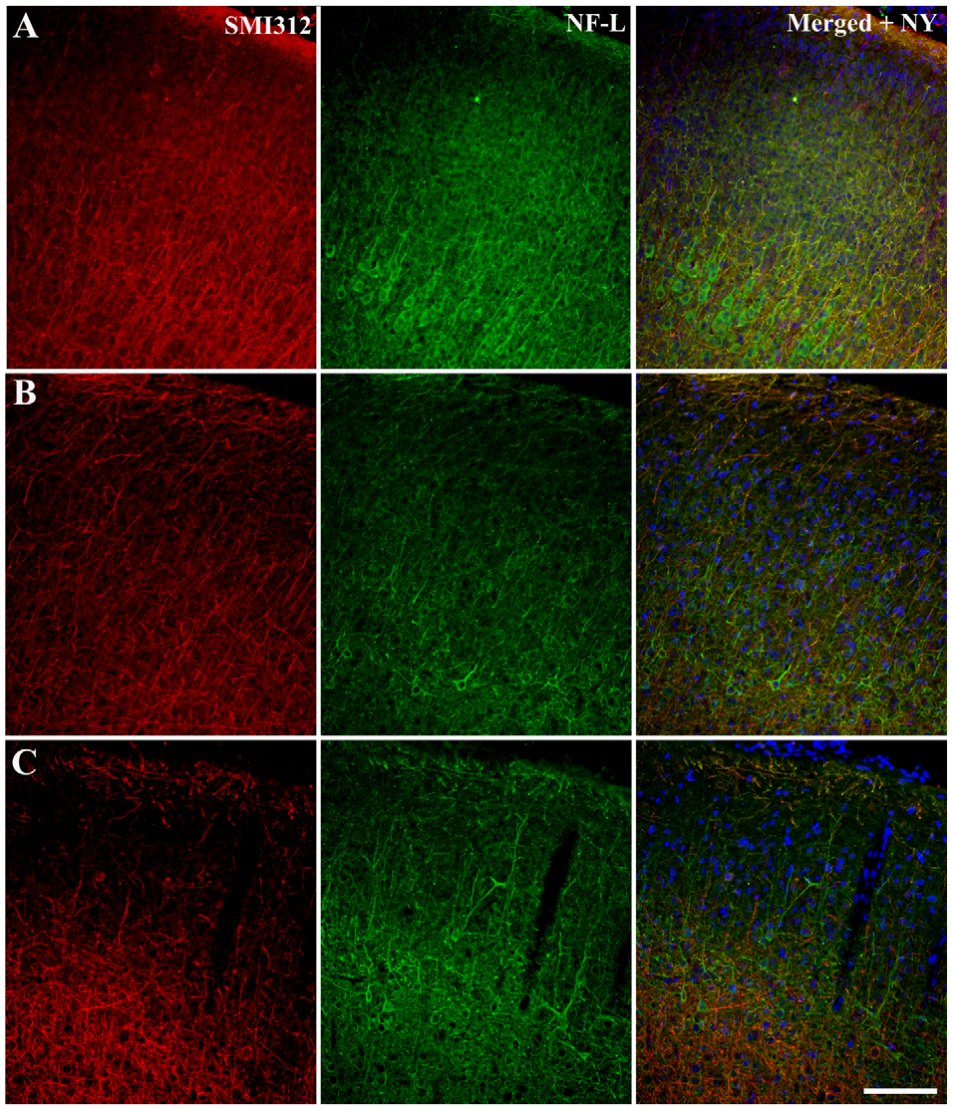
Immunohistochemical labeling of cytoskeletal proteins in acute (non-cultured) brain slices. Acute coronal slices of brain from postnatal (P)4–6 (*A*), 25–28 (*B*) and 50+(*C*) day old mice, cryogenically subsectioned at 40 µm. The images of the neocortex were labeled with cytoskeletal immunohistochemical markers (SMI312 and NF-L) in addition with a nucleus dye (Nuclear yellow) to highlight the structural morphology of neurons. Nuclear yellow staining is pseudo-coloured blue. Scale bar 100 µm.

Changes in neuronal localization and structure during culture were assessed immunohistochemically and compared to that of acute brain slices (Fig. 2). Acute brain slices were not cultured (0 DIV) and allowed to recover for 60 min after slicing, as per reported acute brain slice techniques [28], prior to fixation (Fig. 2). Fixed acute brain slices from P6 animals retained laminar distribution of neuronal cell bodies throughout the cortex with distinct stratification evident at cortical layer V (Fig. 2A). Neurons with a pyramidal morophology in this cortical layer (V) were distinctly immunoreactive for NFL, however, there were no immunolabelled pyramidal cells in layers II/III (Fig. 2A). Immunoreactivity for phosphorylated neurofilaments (SMI312) was present in acute brain slices from all ages (Fig. 2A–C). Acute brain slices from relatively mature animals (P25 and P50+) also displayed distinct neuronal laminar distribution with increased NFL immunoreactivity in cell bodies at cortical layer V but less distinct cellular labeling through layers I–III (Fig. 2B–C). SMI312 immunolabelling was present in neuronal processes of more mature animals, with a lower degree of labeled axons observed in layers II/III compared to that seen at cortical layers V–VI and I–II of slices from P50+ mice (Fig. 2C). It is important to note that we make a comparison between acute brain slices and cultured brain slices. Accordingly, acute brain slice immunoreactivity may be different to that seen in intracardic-fixed mouse brain slices.

**Figure 3:**
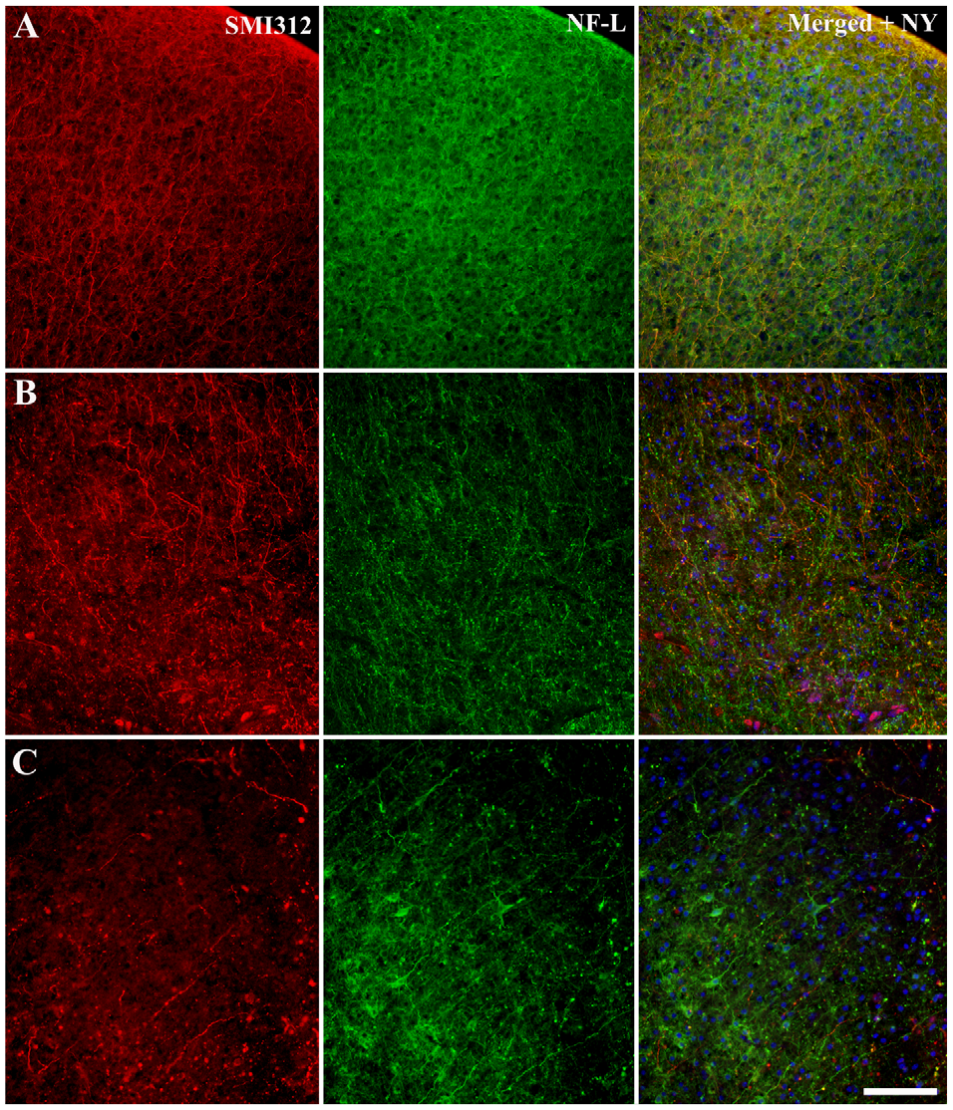
Immunohistochemical labeling of cytoskeletal proteins in cultured brain slices. Organotypic brain slice cultures from P6 (*A*), 25–28(*B*) and 50+(*C*) day old mice, cryogenically subsectioned at 40 µm. Slices were cultured for a period of 7DIV before immunohistochemical analysis with neuronal cytoskeletal markers NF-L and SMI312 in addition with a nucleus dye (Nuclear yellow). Nuclear yellow staining is pseudo-coloured blue. Scale bar 100 µm.

Based on cell viability results detailed above, 7 DIV was selected to be the best time point to compare the neuronal alterations related to culture for slices from all ages. Post-fixation immunohistochemical analysis of P6 brain slices cultured for 7 DIV demonstrated alterations in the pattern of immunolabeling for both NFL and SMI312 throughout the entire neocortex (Fig. 3A). Specifically, labeled neurons did not localize into the distinct layers of the cortex as demonstrated in acute brain slice preparations. The loss of cortical arrangement was particularly evident in cultures from less mature animals, with P50+ slice cultures still retaining the distinct localization of pyramidal neurons at layer V (Fig. 3C). However, the pattern of NFL and SMI312 immunolabeling in mature P50+ acute brain slice preparations did not resemble that seen in cultured brain slice preparations. Specifically, SMI312 immunolabeling was evenly distributed across the entire cortex of brain slices cultured from P6 and P25 animals (Fig. 3A–B), with more stratified labeling of axons in cultured slices from P50+ animals (Fig. 3C). Similarly, cultured P50+ slices also demonstrated clear NFL labeling of pyramidal neurons within cortical layers V and VI. However, it is important to note that there were less pyramidal cells in these long-term culture preparations as compared to acute brain slice preparations.

### 3.3 Culture-induced astrocytic alterations

**Figure 4:**
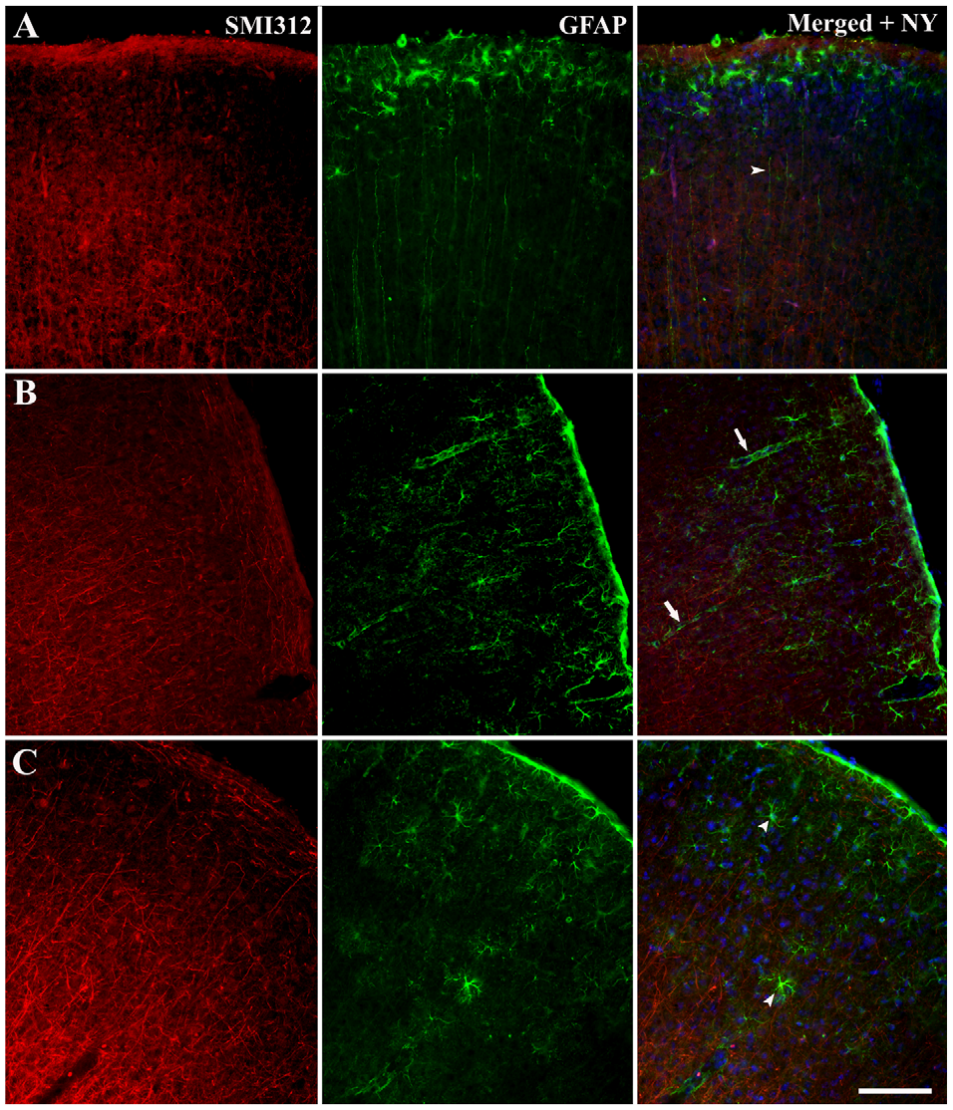
Immunohistochemical labeling of activated astrocytes in acute (non-cultured) brain slices. Acute coronal slices of brain from P6 (*A*), 25–28(*B*) and 50+(*C*) day old mice, cryogenically subsectioned at 40 µm. The images of the neocortex were labeled immunohistochemically with SMI312 and GFAP in addition with a nucleus dye (Nuclear yellow) to investigate astrocytic activation of glial cells. Nuclear yellow staining is pseudo-coloured blue. Scale bar 100 µm.

Alterations in the localization and structure of activated astrocytes (GFAP) were investigated in brain slices cultured for 7 DIV using immunohistochemical techniques. These brain slice cultures were compared to acute slice preparations (Fig. 4). Acute brain slices from P6 animals showed increased GFAP immunoreactivity in cortical layers II and III (Fig. 4A), however, unlike in more mature animals, there was no clear glia limitans on the periphery of cortical layer I (Fig. 4A–C). There was evidence of radial glia through cortical layers II–VI in P6 acute brain slices (Fig. 4A), but this was reduced in P25 and P50+ slices (Fig. 4B–C). It is important to note that in all brain slice preparations, astrocytes were localized into distinct cortical layers.

**Figure 5:**
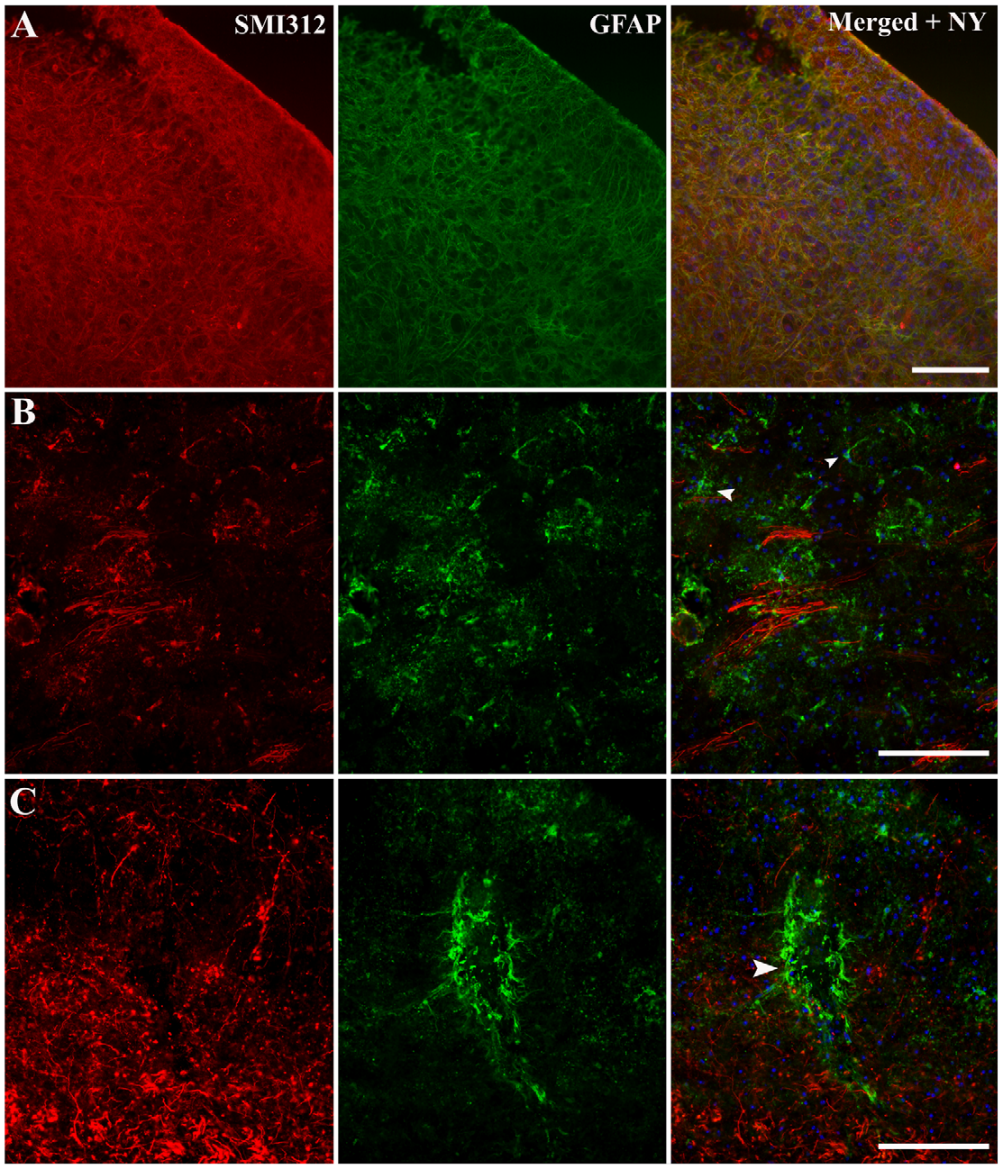
Immunohistochemical labeling of activated astrocytes in cultured brain slices. Organotypic brain slice cultures from P6 (*A*), 25–28(*B*) and 50+(*C*) day old mice, cryogenically subsectioned at 40 µm. Slices were cultured for a period of 7DIV before immunohistochemical analysis with cytoskeletal markers GFAP and SMI312 to identify astrocytic modification during culturing. Images *A* and *B* are higher magnifications. Scale bars 100 µm.

After 7 days of culture, brain slices from P6 mice displayed increased GFAP immunoreactivity across the entire cortex (Fig. 5A). Specifically, although there were increased numbers of GFAP-positive cells, they were not stratified to particular cortical layers as in the acute control slices, and there was no evidence of radial glia (Fig. 5A). The level of GFAP expression was significantly lower in more mature cultured brain slices, with patches of GFAP-positive cells observed across the cortex (Fig. 5B–C). In P50+ cultured brain slices, these GFAP-positive cells formed small clusters resembling astrocytic end-feet around blood vessels (Fig. 5C). Indeed, at higher magnification (Fig. 5B–C), GFAP immunoreactivity is localized to regions of increased SMI312 immunolabeling with these labeled cells having a abnormal structural appearance (Fig. 5B–C).

**Figure 6:**
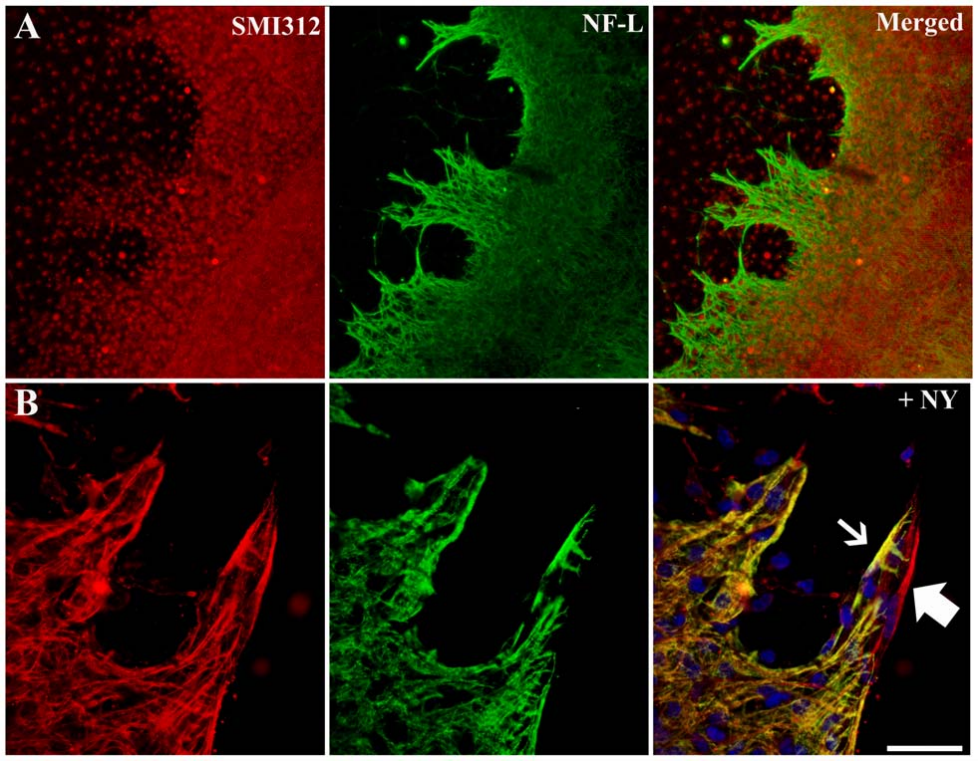
Co-localization of glial and cytoskeletal immunolabeling in neonatal brain slices cultured for 14 DIV. The periphery of organotypic brain slice cultures from P6 animals cultured for 14 DIV and double-immunolabeled with SMI312 (red; *A*, *B*) and either NFL (green; *A*) or GFAP (green; *B*) in addition with a nucleus dye (Nuclear yellow). Nuclear yellow staining is pseudo-coloured blue. Slice protrusions contain cells that are immunoreactive for either neuronal proteins (thick-arrow) only or both neuronal and glial proteins (thin-arrow). Scale bar 50 µm.

As shown in the cell viability assays, P6 cultured brain slices were able to survive culture for longer time periods, with optimal cell viability and reduced death observed at 14 DIV. However, by 14 DIV, there were cellular protusions from the edges of cultured slices as well as individual cells (Fig. 6). Interestingly, these extensions onto the substrate contained cells that expressed both GFAP and SMI312 (Fig. 6B). These cells did not have growth guidance features (growth cones) and did not display the stereotypical neuronal morphology (i.e neurite polarization).

### 3.4 Protein expression changes during brain slice culture

**Figure 7:**
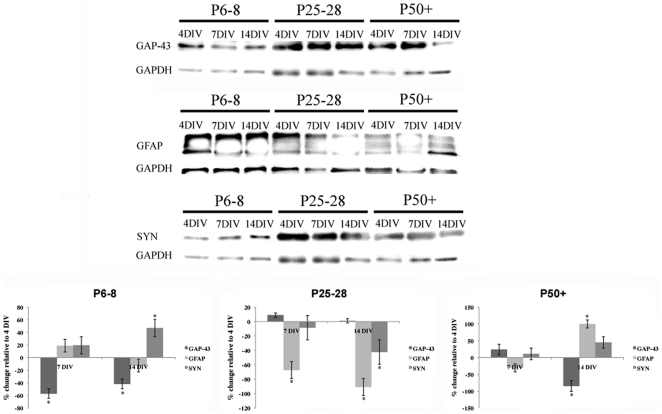
Western blot analysis of organotypic brain slice cultures. The protein levels of GAP-43, GFAP, and synaptophysin (SYN) in brain slice cultures from P6, 25–28 and 50+ were assessed after 4, 7, and 14 days culture. The proteins changes are presented as a percentage of change compared to 4 DIV. Please note that statistical significance was calculated in cultures derived from animals of the same age and not across the different age groups. *p<0.05.

The expression profile of GAP-43, synaptophysin and GFAP during organotypic brain slice culture was investigated. In slices from P6 animals, there was significant decrease (p<0.05) in the expression of GAP-43 over 14 DIV (Fig. 7). Conversely, a significant increase in the expression of synaptophysin was also observed during the culture period. As indicated with immunohistochemical markers, there was no significant change in the expression of GFAP over 14 days of culture (Fig. 7). The expression of GFAP in both P25 and P50+ brain slice cultures was significantly reduced after 7 DIV, however, there was an increase in GFAP expression in P50+ cultures after 14 DIV (Fig. 7). GAP-43 was significantly reduced in P25 brain slices cultured over 14 DIV, with no significant alterations in GAP-43 in P50+ cultures. Synaptophysin expression did not significantly change in mature cultures during the entire culture period (Fig. 7). It is critical to note that changes in expression of proteins were only determined in cultures derived from animals of the same age. No statistical comparisons were made between aged groups.

## Discussion

We show in this study the major advantages and limitations of using neonatal and mature brain tissue for long-term organotypic whole brain slice culture. We illustrate that neonatal mouse tissue survives long-term culture significantly better than tissue from more mature animals, and, after 14 DIV, proteins associated with a mature phenotype are expressed. However, the neuronal and glial organization of the cultured neonatal cortex is significantly different to that of mature brain slices, with increased expression of glial proteins throughout the cortex, loss of patterns of neuronal localization within distinct layers, no distinct glia limitans and the presence of what may be progenitor-like cells forming extensions from the periphery of the slice.

Organotypic brain slice cultures are extensively used to investigate mechanistic insights into neuro- development, transmission and degeneration [11]. The major limitation of organotypic brain slice culture techniques is determining the relative maturity of slices or to what extent cultured slices resemble that of living tissue *in situ*. In this regard, cultures are usually derived from neonatal tissue and maintained for an extended culture period [8]. The rationale for culturing neonatal tissue is that the nerve cells survive explantation more readily as they are less vulnerable to hypoxic-ischemic damage [8], [29], [30]. The results of the current study support this proposition illustrating that brain tissue derived from younger mice are significantly less vulnerable in long-term culture. For the pruposes of this study, we implemented a single culturing protocol for both neonatal and mature brain tissue. Previous studies, albeit related to hippocampal preparations, have suggested the possible benefits of increasing the oxygenation of dissecting medium as the mature brain is more vulnerable to hypoxic damage [31].

Relatively few studies have attempted to culture adult tissue, with only a limited number of investigations having success using rat hippocampal tissue from P20–25 animals [13], [31], [32]. Indeed, these studies focused mainly on the retention of maturity-associated electrophysiological properties possibly in response to comparative studies showing maturation of synaptic responses occur more rapidly in cultures prepared from older animals compared to younger animals [14]. Interestingly, these techniques have not been attempted using mature mouse tissue, which would be particularly useful given the increasing availability of a range of transgenic mouse lines. Very few studies have attempted to culture whole brain slices from young adult mice, and in these circumstances the slices were mainly utilized as a substrate to culture dissociated cells [15]. In the current study, we demonstrate an age-dependant increase in cellular vulnerability when culturing whole brain slices in mice. However, we do show that mature slice cultures from P25 mice survive for 7 DIV, which are well suited for short-term studies and may be advantageous for experiments involving chronic stimulation or implanted recording electrodes for multi-site recording of electrical activity. Given cellular maturity and reduced morphological alterations during culture, P25 cultures could be an ideal extension of current acute brain slice techniques. It is important to note that although there are fewer morphological alterations in the cortex of more mature cultures, this needs to be further validated functionally using electrophysiological techniques. Additionally, there are constant improvements in markers of neuronal death/apoptosis/necrosis, which may prove useful in validating current results [21]. In this study PI was utilized as a nuclei stain which would be indicative of membrane damage. Although this cell impermeant stain is by far the most commonly used fluorescent probe, it cannot be used to distinguish between necrosis and apoptosis. The results of AlamarBlue ™ needs further qualification, particularly in P6 cultures, as increased signal may be indicative of cell proliferation over time and mask results on cell survival. Indeed, previous studies have shown increased cell proliferation in brain slice cultures [21].

We observed a time-dependant increase in the expression of synaptophysin in neonatal brain slice cultures over 14 DIV. This finding may be indicative of a gradual shift in these cultured brain slices to a mature phenotype. This is further supported by a significant and concomitant decrease in the expression of the growth-associated proteins GAP-43. There were no similar increases in synaptophysin expression in slices from mature animals, with the decrease in synaptophysin expression during culture temporally associated with the progressive death of cells over an extended culture period. Previous ultrastructural studies in rat organotypic hippocampal slice cultures have illustrated a significant increase in the density of synaptic contacts at 21 DIV, which is approximately double that observed at 7 DIV [33]. Additionally, earlier work has shown that synaptic activity steadily increases during the first few weeks of organotypic hippocampal slice culture [34], [35], which may be due to variables such as connective reorganization, synaptic protein expression and dendritic spine density. It is proposed that as neonatal organotypic hippocampal cultures display increased synaptogenesis over time, then increased periods of culture would result in physiological conditions similar to that seen *in vivo*, and different to that observed in acute brain slice cultures [11]. However, no significant difference was noted in the electrophysiological properties of hippocampal organotypic cultures and acute brain slices [9], [36].

Neonatal brain cultures lose the distinct cytoarchitectural cellular arrangements present *in vivo* and in acute brain slices of mature animals. Acute brain slices have a distinct pattern of SMI312 and NFL immunolabelling depending on developmental stage. The expression profiles of cultured slices from adult animals are similar to that previously reported both in rats and guinea pigs [22], [37], [38]. Specifically, SMI312 is found mainly in pyramidal cell populations (axonal domain) of the neocortex of mature animals (as shown in acute control slices), and has been proposed to have a role in myelination. This highlights the critical importance of neonatal age when investigating the neocortex in organotypic brain slice cultures. Indeed, GABAergic interneuronal lineages selectively sort into specific cortical layers during early postnatal development [17]. Specifically, in rodents, the majority of interneurons are generated from the ventral telencephalon and are found to occupy primarily the deep and superficial cortical areas during embryonic development [39], [40]. These cells migrate through the cortex during the early (P1) postnatal period and only after this stage do they sort into the appropriate cortical layers with correct laminar distribution of these cells seen in layer II/III after P7 [17]. As the cultures from this study were derived from P4 animals, it is possible that there is a significant disruption to the migration of these cells into their correct laminar distributions. There may be a number of reasons for the disruption of cortical neuronal migration and integration into the correct layers in these *in vitro* preparations, including loss or abnormal release of growth factors during explantation. In addition, this study determined that there was increased levels of activated glia throughout the entire cortex in cultured brain slices derived from neonatal cultures. We also showed in this study that radial glia are prominent in our acute neonatal brain slice preparations, and it is possible that culturing conditions stimulate these cells to differentiate into astrocytes [41]. The disruption of the radial glia, which occurs early in neonatal cultures (7 DIV), may thus have significant consequences for neuronal migration in the developing brain. New neurons reach their cortical layers in the developing cortex either by somal translocation whereby a neuron cell body travels within a pial contacting radial process [42], or by locomotion, whereby the cells migrate along radial glia [43]. Indeed, in this study we see the loss of not only the radial glia but also of the *glia limitans*. Further ultrastructural studies would look at the dendritic re-organizations that are associated with long-term culture as this study mainly focused on axonal alterations.

At 14 DIV, organotypic brain slice cultures from neonatal mice also displayed cellular protrusions from the edges of the cortex. Although the morphology of these cellular protrusions resembled glial cells, they also expressed neuronal proteins. Similar neuritic processes extending onto the substrate have been reported [12], [33]. In these studies, the processes were proposed to constitute a rudimentary ‘root’ system that functioned to not only maintain the slice on the membrane but to also to provide more efficient medium access [12]. However, these processes may be indicative of aberrant three-dimensional plasticity that would otherwise be prevented in mature cultures by the presence of a *glia limitans* at the periphery of the slice. Importantly, this process further highlights the increased presence of possibly progenitor-like cells in slice cultures at 14 DIV, which may significantly affect any experimental data subsequently obtained. It is important to however further confirm the presence of these progenitor cells using specific markers (NG2, nestin etc.).

In summary, we illustrate the cortical neuronal and glial alterations in organotypic whole brain slice cultures from mice of varying maturity. Our results demonstrate that organotypic cultures from neonatal mouse tissue survive longer, however, do not retain the cellular and cytoarchitectural features of mature tissue. Additionally, cultures from neonatal animals have persistently higher levels of astrocytic activation and the continued presence of possible progenitor-like cells giving rise to ectopic growth.

## Supporting Information

Figure S1 **Example of PI staining in acute (0DIV) and cultured brain slices from P50+ mice.**
(TIF)

Figure S2 **Example of PI staining in acute (0DIV) and cultured brain slices from P25–28 mice.**
(TIF)

Figure S3 **Example of PI staining in acute (0DIV) and cultured brain slices from P6 mice.**
(TIF)

Figure S4 **Approximation of culture slice area used in experiment.** Coronal slices were obtained from within the sections marked with black lines. The red line indicates line through bregma.
(TIF)
