# Supplementary figures and images for: Characterization of Cortical Neuronal and Glial Alterations during Culture of Organotypic Whole Brain Slices from Neonatal and Mature Mice
Source: PLoS One. 2011 Jul 15;6(7):e22040. doi: 10.1371/journal.pone.0022040 (PMC3137607; doi:10.1371/journal.pone.0022040)

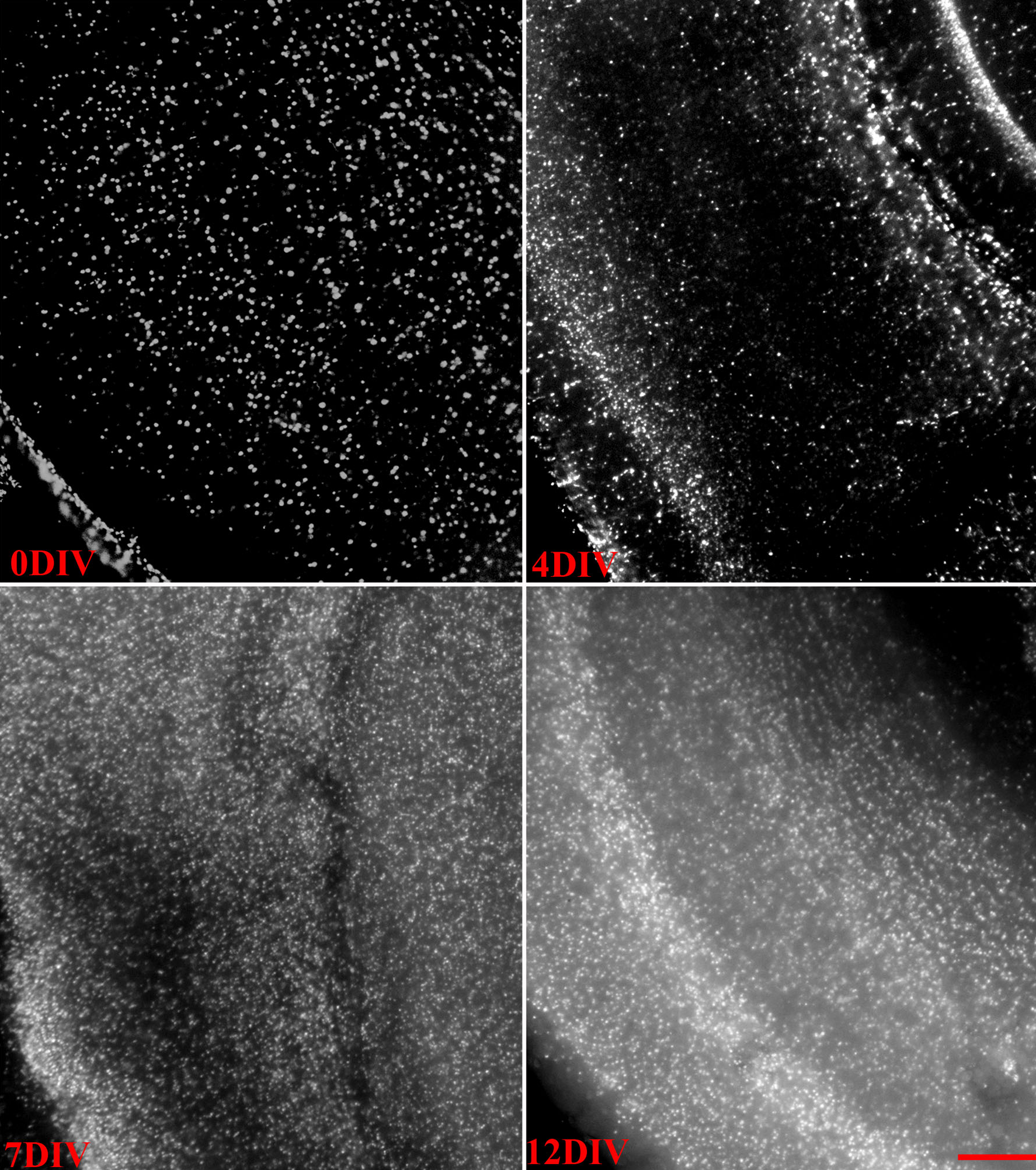

Supplement: Figure S1 — Example of PI staining in acute (0DIV) and cultured brain slices from P50+ mice. (TIF) [file pone.0022040.s001.tif]

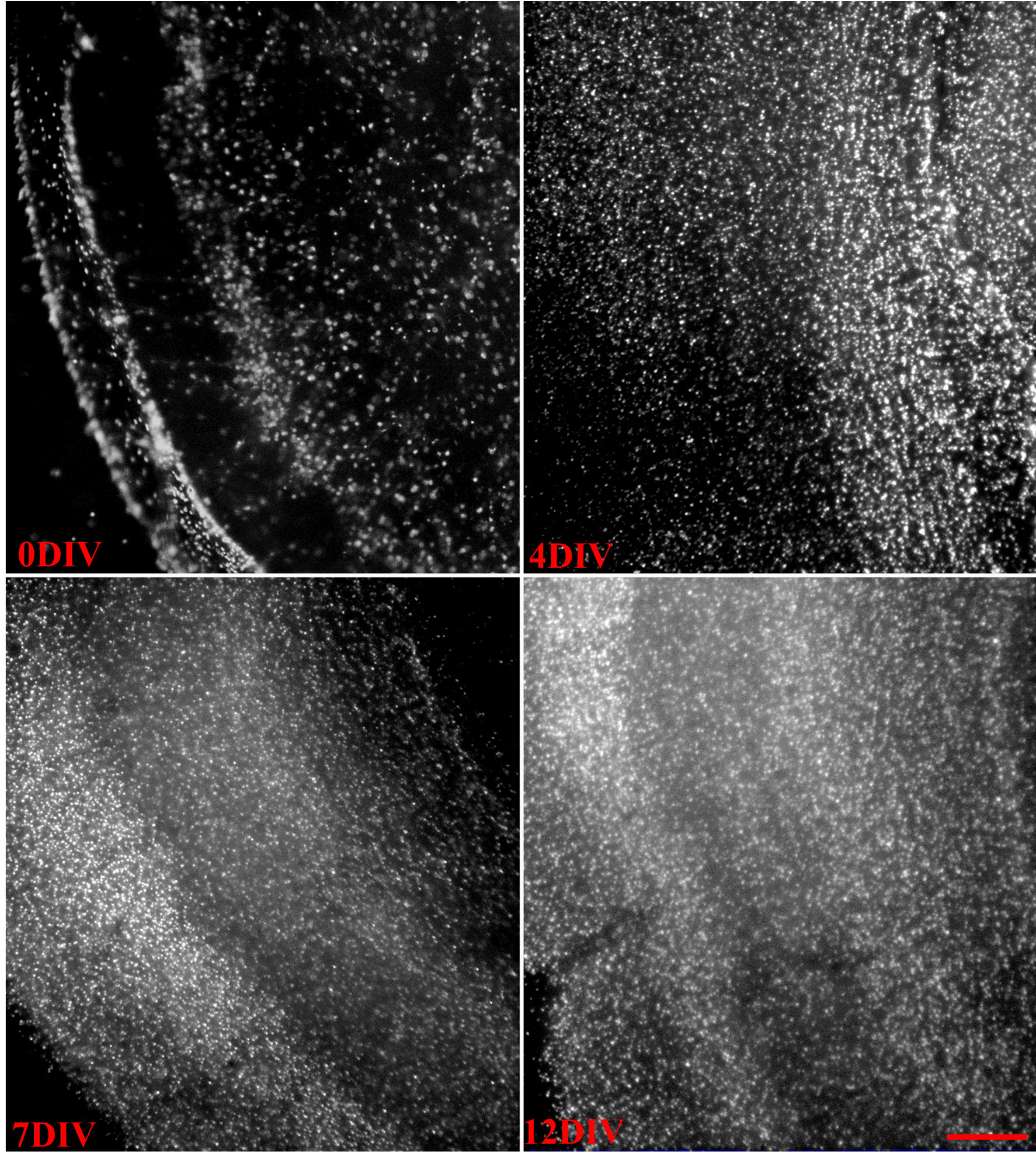

Supplement: Figure S2 — Example of PI staining in acute (0DIV) and cultured brain slices from P25–28 mice. (TIF) [file pone.0022040.s002.tif]

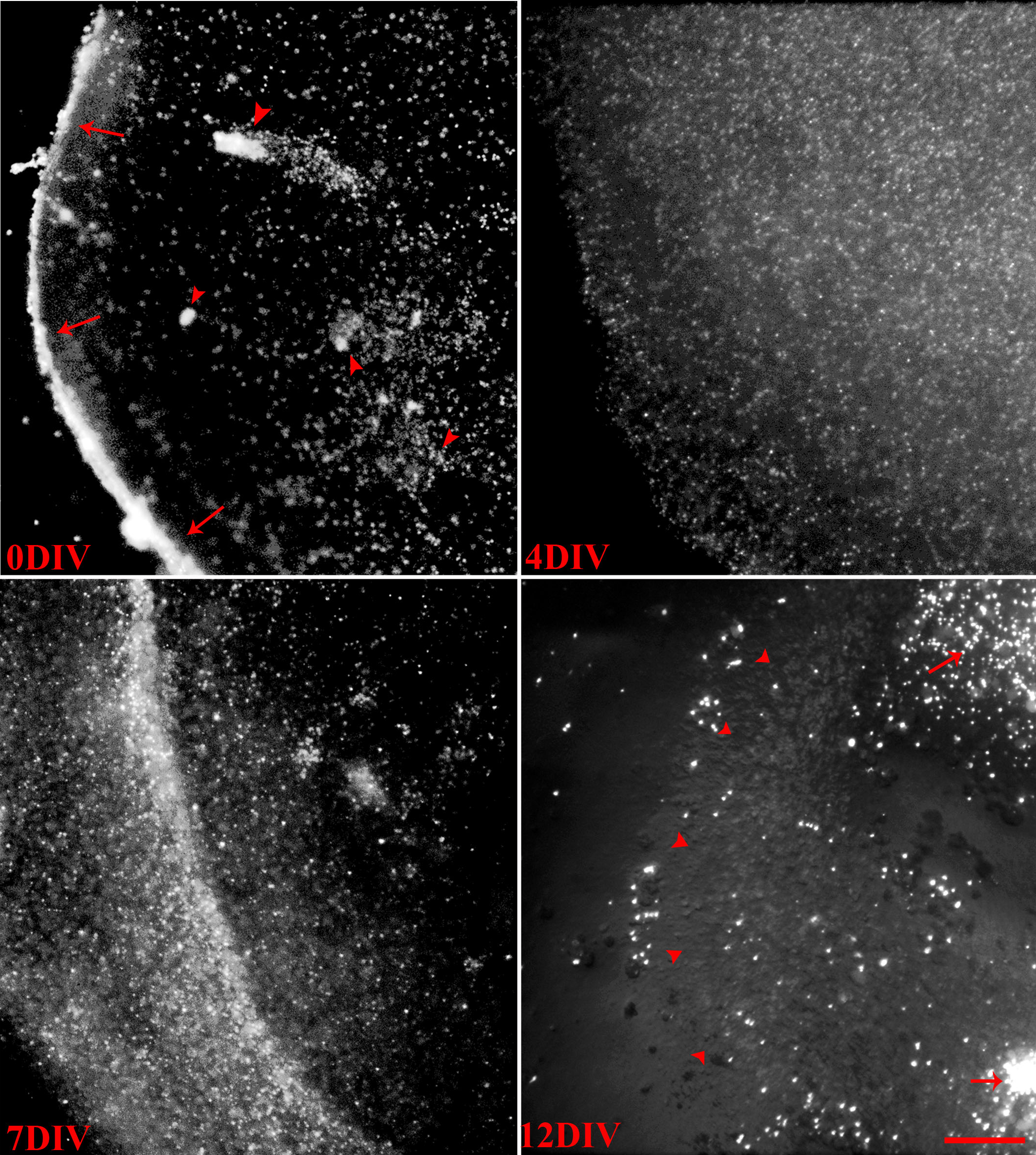

Supplement: Figure S3 — Example of PI staining in acute (0DIV) and cultured brain slices from P6 mice. (TIF) [file pone.0022040.s003.tif]

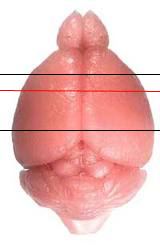

Supplement: Figure S4 — Approximation of culture slice area used in experiment. Coronal slices were obtained from within the sections marked with black lines. The red line indicates line through bregma. (TIF) [file pone.0022040.s004.tif]
